# The measurement and correlation analysis of scleral and choroid thickness in branch retinal vein occlusion

**DOI:** 10.1038/s41598-024-65111-3

**Published:** 2024-06-23

**Authors:** Xiao Yu, Yuling Zou, Ziqing Mao, Huimin Fan, Xiaolong Yu, Teng Liu, Zhipeng You

**Affiliations:** The Affiliated Eye Hospital, Jiangxi Medical College, Nanchang University, Nanchang, 330006 China

**Keywords:** Branch retinal vein occlusion, Choroid thickness, Scleral thickness, Oncogenesis, Retinal diseases

## Abstract

To use Optical Coherence Tomography (OCT) to measure scleral thickness (ST) and subfoveal choroid thickness (SFCT) in patients with Branch Retinal Vein Occlusion (BRVO) and to conduct a correlation analysis. A cross-sectional study was conducted. From May 2022 to December 2022, a total of 34 cases (68 eyes) of untreated unilateral Branch Retinal Vein Occlusion (BRVO) patients were recruited at the Affiliated Eye Hospital of Nanchang University. Among these cases, 31 were temporal branch vein occlusions, 2 were nasal branch occlusions, and 1 was a superior branch occlusion. Additionally, 39 cases (39 eyes) of gender- and age-matched control eyes were included in the study. Anterior Segment Optical Coherence Tomography (AS-OCT) was used to measure ST at 6 mm superior, inferior, nasal, and temporal to the limbus, while Enhanced Depth Imaging Optical Coherence Tomography (EDI-OCT) was used to measure SFCT. The differences in ST and SFCT between the affected eye, contralateral eye, and control eye of BRVO patients were compared and analyzed for correlation. The axial lengths of the BRVO-affected eye, contralateral eye, and control group were (22.92 ± 0.30) mm, (22.89 ± 0.32) mm and (22.90 ± 0.28) mm respectively, with no significant difference in axial length between the affected eye and contralateral eye (P > 0.05). The SFCT and ST measurements in different areas showed significant differences between the BRVO-affected eye, contralateral eye in BRVO patients (P < 0.05). The CRT of BRVO-affected eyes was significantly higher than that of the contralateral eyes and the control eyes (P < 0.001). In comparison between BRVO-affected eyes and control eyes, there were no statistically significant differences in age and axial length between the two groups (P > 0.05). However, significant differences were observed in SFCT and temporal, nasal, superior, and inferior ST between the two groups (P < 0.05). The difference in temporal ST between the contralateral eyes and the control eyes was not statistically significant (t = − 0.35, P = 0.73). However, the contralateral group showed statistically significant increases in SFCT, nasal, superior and inferior ST compared to control eyes (t = − 3.153, 3.27, 4.21, 4.79, P = 0.002, 0.002, < 0.001, < 0.001). However, the difference between the CRT of the contralateral and control eyes was not statistically significant (P = 0.421). When comparing SFCT and ST between BRVO-affected eyes with and without macular edema, no statistically significant differences were found (t = − 1.10, 0.45, − 1.30, − 0.30, 1.00; P = 0.28, 0.66, 0.21, 0.77, 0.33). The thickness of SFCT and temporal ST in major BRVO group is higher than the macular BRVO group and the difference was statistically significant (t = 6.39, 7.17, P < 0.001 for all). Pearson correlation analysis revealed that in BRVO patients, there was a significant positive correlation between SFCT/CRT and temporal ST (r = 0.288, 0.355, P = 0.049, 0.04). However, there was no correlation between SFCT/CRT and nasal ST, superior ST, and inferior ST (P > 0.05). In BRVO patients, both SFCT/CRT and ST increase, and there is a significant correlation between SFCT/CRT and the ST at the site of vascular occlusion.

## Introduction

Retinal Vein Occlusion (RVO) refers to the blockage of the retinal vein system due to various reasons, leading to a common retinal vascular disease characterized by retinal ischemia and hypoxia. Depending on the degree of retinal vessel involvement, RVO can be further classified into Central Retinal Vein Occlusion (CRVO) and Branch Retinal Vein Occlusion (BRVO)^1^. The global prevalence of BRVO in people aged 30–89 years was 0.64%, making it the most common type of RVO, and it is associated with various factors such as hypertension and atherosclerosis^2^. BRVO often occurs at arteriovenous crossings and is mainly related to changes in hemodynamics, vascular endothelial cell damage, and hypercoagulability, although the exact mechanism of its occurrence is not clear^3^. Recent studies have shown that the pathology of RVO involves not only the retina but also the choroid. Aribas et al. found that in RVO, the total number of choroidal vessels and the capillary blood flow density in the choroid are reduced, while the choroidal large vessel layer shows vascular dilation^4^. Previous studies have indicated that compared to the normal control group, Subfoveal Choroid Thickness (SFCT) increases in RVO eyes, and SFCT decreases after intravitreal anti-vascular endothelial growth factor injection therapy, suggesting that the increase in SFCT is associated with an increase in vascular endothelial growth factor in the choroid, changes in vascular permeability, and fluid leakage^5–7^. Meryem et al. demonstrated that in BRVO patients, SFCT increases, the area of intercellular stroma and total choroidal area increase, while the total choroidal blood flow does not change, suggesting that the increase in SFCT in BRVO patients is due to extracellular fluid flowing from the retina into the choroid, causing choroidal interstitial edema, rather than changes in vascular quantity and density^8^. Similarly, previous studies have suggested that changes in the choroid in RVO may be due to alterations in choroidal hemodynamics, rather than changes in vascular quantity^4,9^. Recent research has indicated that RVO shares similar pathogenic mechanisms with pachychoroid disease^10^. Venkatesh et al. proposed that an increase in scleral thickness or hardness may be a possible pathogenic mechanism of pachychoroid disease^11^. Whether Scleral Thickness (ST) also plays a significant role in the occurrence of RVO is a question that has been rarely investigated. Therefore, we utilized Anterior Segment Optical Coherence Tomography (AS-OCT) to study changes in ST and SFCT in BRVO and conducted correlation analysis, aiming to provide new insights into the etiology and treatment of BRVO in clinical practice.

## Research methods

### General information

This was a cross-sectional study. A total of 34 untreated unilateral BRVO patients (68 eyes) who visited our hospital from May 2022 to December 2022 were selected. Additionally, 39 normal control subjects with gender and age matched to the unilateral BRVO eyes were included in the study. Patients with a history of intraocular surgery, concomitant glaucoma, uveitis, idiopathic polypoidal choroidal vasculopathy, age-related macular degeneration, central serous chorioretinopathy, myopia, neurodegenerative diseases, prior intravitreal injections, laser treatment, hypertension which was the absence of a previous history of hypertension and a blood pressure measurement at presentation that did not meet the diagnostic criteria for hypertension guidelines, diabetes, connective tissue diseases, and those with unclear imaging data of systemic diseases were excluded. All patients enrolled in this study underwent AS-OCT, Enhanced Depth Imaging Optical Coherence Tomography (EDI-OCT), Best Corrected Visual Acuity (BCVA) assessment, fundus fluorescein angiography, axial length measurement, intraocular pressure measurement, slit lamp examination, and dilated fundus examination between 9 and 11 am. The study was conducted in accordance with the Helsinki Declaration and approved by the Affiliated Eye Hospital of Nanchang University's ethics committee (YLP20211210). All patients provided informed consent and signed the informed consent form.

### Imaging examinations

**Figure 1:**
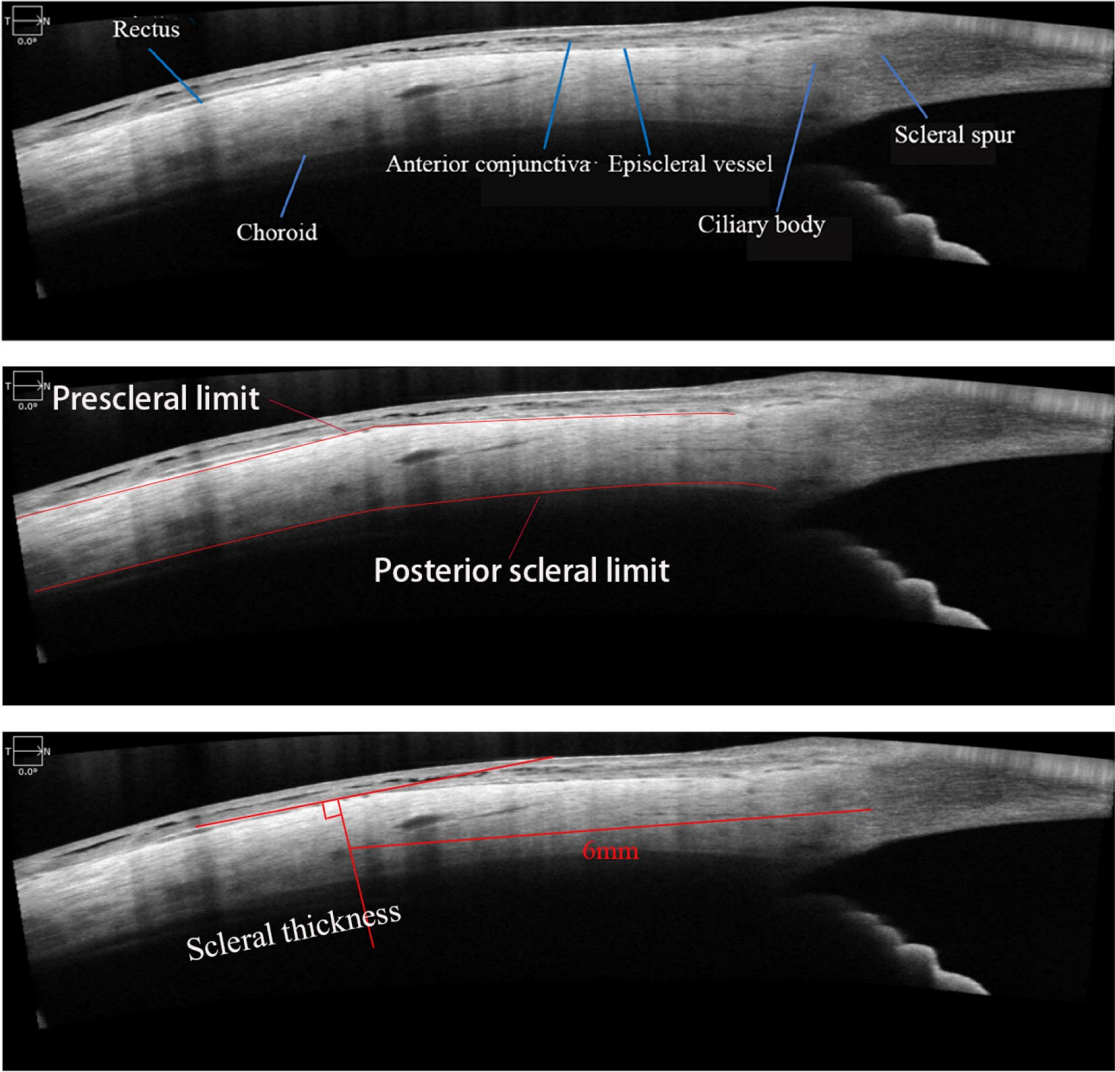
The measurement of scleral thickness.

**Figure 2:**
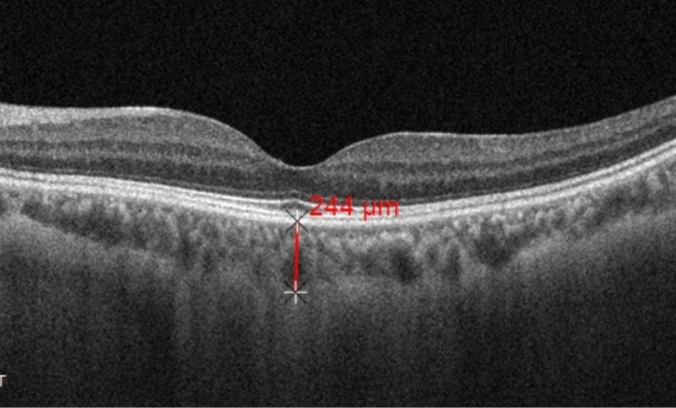
The measurement of subfoveal choroid thickness.

The patients underwent AS-OCT and EDI-OCT using ZEISS CIRRUS HD^5000^. AS-OCT measurements were taken 6 mm posterior to the limbus in the superior, inferior, nasal, and temporal directions to assess ST. According to Suzuki et al., the four straight muscles are adjacent to the sclera and appear as low-reflectance lines^12^. The scleral anterior border was determined by the boundary between the low-reflectance straight muscles (superior rectus, inferior rectus, medial rectus, lateral rectus) and the high-reflectance sclera, while the scleral posterior border was determined by the signal from the choroid (Fig. 1). The ST measurements were conducted following the method described by Read et al.^13–15^. The ST in the four directions represented by the vertical distance between the anterior and posterior borders of the sclera 6 mm posterior to the limbus. EDI-OCT was performed between 9 and 11 am to measure SFCT. The SFCT was defined as the vertical distance from Bruch's membrane to the inner surface of the sclera in the central foveal region, represented by the vertical distance between the high-reflectance line below the retinal pigment epithelium and the low-reflectance line on the inner surface of the sclera (Fig. 2). ST and SFCT measurements were taken three times by the same ophthalmologist, and the average value was recorded and analyzed. Axial length measurements were conducted for all patients using the IOL Master from ZEISS MASTER 5.5.

### Statistical methods

Statistical analysis was performed using SPSS 24.0 software. For continuous data, the Shapiro–Wilk test was used to assess normality, and Levene's test was used to test for homogeneity of variance. As the data followed a normal distribution and exhibited homogeneity of variance, independent samples t-test was used for comparisons between two groups, and one-way analysis of variance (ANOVA) was used for comparisons among multiple groups. Pearson's correlation analysis was conducted to assess correlation analysis. Continuous data are presented as mean ± standard deviation (mean ± SD). For categorical data comparisons, the chi-square test or Fisher's exact test was used. A significance level of P < 0.05 was considered statistically significant.

## Results

### General information

The BRVO group consisted of 34 patients with 34 affected eyes, including 18 females and 16 males. There were 20 cases in the left eye and 14 cases in the right eye, with 31 patients having temporal branch vein occlusion, 2 patients with nasal branch occlusion, and 1 patient with superior branch occlusion. The average age was (57.38 ± 12.82) years, with a disease duration of (4.67 ± 1.87) months. The axial length of the affected eyes was (22.92 ± 0.30) mm, ranging from 22.43 to 23.53 mm, while the axial length of the contralateral eyes was (22.89 ± 0.32) mm, ranging from 22.26 to 23.48 mm. The difference in axial length between BRVO-affected eyes and contralateral eyes was not statistically significant (t = 0.37, P = 0.71).

The control group comprised 39 participants with 39 eyes, including 23 females and 16 males, with 20 left eyes and 19 right eyes. The average age was (57.90 ± 7.83) years, and the axial length was (22.90 ± 0.28) mm, ranging from 22.31 to 23.67 mm. There were no statistically significant differences in age and axial length between the BRVO group and the control group (t = − 0.21, 0.34; P = 0.83, 0.74) (Table 1). The differences of SFCT, temporal, nasal, superior and inferior ST among the three groups were statistically significant (F = 144.34, 18.03, 57.40, 70.00, 62.55, all P values were less than 0.001). Multiple comparisons indicate that BRVO-affected eyes showed statistically significant increases in SFCT, temporal, nasal, superior and inferior ST compared to contralateral eyes and the control group eyes (P < 0.05). The CRT of BRVO-affected eyes was significantly higher than that of the contralateral eyes and the control eyes (P < 0.001). The difference between the CRT of the contralateral and control eyes was not statistically significant (P = 0.421).

**Table 1:** Comparison of general information among the three groups.

Category	BRVO-affected	Contralateral eyes	Control eyes	P_1_	P_2_	P_3_
Axial lengths (mm)	22.92 ± 0.30	22.89 ± 0.32	22.90 ± 0.28	0.71	0.74	0.94
Age (years)	57.38 ± 12.82	57.38 ± 12.82	57.90 ± 7.83	–	0.83	–
Sex						
Female	18	18	23	–	0.60	–
Male	16	16	16	–	0.60	–

*BRVO* branch retina vein occlusion, *P1* statistical values for comparisons between BRVO-affected and contralateral eyes, *P2* statistical values for comparisons between BRVO-affected and control eyes, *P3* statistical values for comparisons between contralateral eyes and control eyes.

P < 0.05 was statistically significant.

### Comparison between BRVO-affected eyes and contralateral eyes

The Best Corrected Visual Acuity (BCVA) of BRVO-affected eyes was (0.92 ± 0.41) LogMAR, while that of contralateral eyes was (0.08 ± 0.73) LogMAR. BRVO-affected eyes showed statistically significant increases in SFCT, temporal, nasal, superior and inferior ST compared to contralateral eyes (P < 0.05) (Table 2).

**Table 2:** Comparison between BRVO-affected eyes, contralateral eyes and control eyes.

Category	BRVO-affected eyes	Contralateral eyes	Control eyes	t_1_	t_2_	t_3_	*P*_*1*_	*P*_*2*_	*P*_*3*_
SFCT (μm)	391.88 ± 38.70	246.18 ± 18.13	235.41 ± 10.50	19.88	24.27	− 3.15	< 0.001*	< 0.001*	0.002
Temporal ST (μm)	447.88 ± 50.55	388.62 ± 29.76	384.67 ± 60.55	5.89	4.80	− 0.35	< 0.001*	< 0.001*	0.73
Nasal ST (μm)	466.38 ± 47.24	357.82 ± 25.03	389.28 ± 51.00	11.84	6.67	3.27	< 0.001*	< 0.001*	0.002
Superior ST (μm)	478.44 ± 53.43	346.59 ± 28.70	389.69 ± 53.23	12.68	7.09	4.21	< 0.001*	< 0.001*	< 0.001*
Inferior ST (μm)	455.32 ± 49.08	341.47 ± 27.00	385.18 ± 46.82	11.86	6.24	4.79	< 0.001*	< 0.001*	< 0.001*

*BRVO* branch retina vein occlusion, *SFCT* subfoveal choroid thickness, *ST* scleral thickness.

t_1_ and P_1_: statistical values for comparisons between BRVO-affected and contralateral eyes; t_2_ and P_2_: statistical values for comparisons between BRVO-affected and control eyes;t_3_ and P_3_: Statistical values for comparisons between contralateral eyes and control eyes; P < 0.05 was statistically significant; * indicates P < 0.001.

### Comparison between BRVO-affected eyes and the control group

The BCVA of BRVO-affected eyes was (0.92 ± 0.41) LogMAR, while that of the control group was (0.07 ± 0.68) LogMAR. Statistical analysis revealed significant differences in SFCT, temporal ST, nasal ST, superior ST, and inferior ST between the two groups (t = 24.27, 4.80, 6.67, 7.09, 6.24; P < 0.001 for all) (Table 2).

### Comparison between the control group and contralateral eyes

The difference in axial length between the control group and contralateral eyes was not statistically significant (t = 0.08, P = 0.94). The difference in temporal ST between the contralateral eyes and the control eyes was not statistically significant (t = − 0.35, P = 0.73).

However, the contralateral group showed statistically significant increases in SFCT, nasal, superior and inferior ST compared to control eyes (t = − 3.153, 3.27, 4.21, 4.79, P = 0.002, 0.002, < 0.001, < 0.001) (Table 2).

### Comparison between major BRVO and macular BRVO

Patients with BRVO were further divided into more pronounced venous congestion group (major BRVO) and less congestion group (macular BRVO) according to whether the occlusion site involved the macular blood vessels. The comparison of nasal, superior, and inferior ST between the major BRVO group and macular BRVO group did not show any statistically significant differences (t = 0.92, 0.81, 1.05, P = 0.37, 0.43, 0.30). However, the thickness of SFCT and temporal ST in major BRVO group is higher than the macular BRVO group and the difference was statistically significant (t = 6.39, 7.17, P < 0.001 for all) (Table 3).

**Table 3:** Comparison between major BRVO and macular BRVO.

Category	Major BRVO	Macular BRVO	t	P
Age (years)	57.50 ± 10.54	57.25 ± 15.35	0.06	0.96
Axial lengths (mm)	22.90 ± 0.31	22.94 ± 0.31	-0.33	0.74
SFCT (μm)	536.17 ± 27.44	481.19 ± 22.04	6.39	< 0.001*
Tempora ST (μm)	484.78 ± 35.09	406.38 ± 25.99	7.17	< 0.001*
Nasal ST (μm)	473.39 ± 47.70	458.50 ± 46.97	0.92	0.37
Superior ST (μm)	485.44 ± 62.00	470.56 ± 42.43	0.81	0.43
Inferior ST (μm)	463.67 ± 43.48	445.94 ± 54.58	1.05	0.30

*BRVO* branch retina vein occlusion, *SFCT* subfoveal choroid thickness, *ST* scleral thickness.

P < 0.05 was statistically significant; * indicates P < 0.001.

### Comparison between BRVO-affected eyes with and without macular edema

Among the BRVO-affected eyes, 20 eyes had macular edema (ME group) and 14 eyes did not have ME (non-ME group). The axial length of the ME group was (22.834 ± 0.25) mm, while that of the non-ME group was (23.045 ± 0.34) mm. The difference was not statistically significant (t = − 1.96, P = 0.06). The comparison of SFCT and temporal, nasal, superior, and inferior ST between the ME group and non-ME group did not show any statistically significant differences (t = − 1.10, 0.45, -1.30, -0.30, 1.00; P = 0.28, 0.66, 0.21, 0.77, 0.33).

### Correlation analysis between SFCT/CRT and ST

Pearson correlation analysis revealed that in BRVO patients, there was a significant positive correlation between SFCT and temporal ST (r = 0.288, P = 0.049). However, there was no correlation between SFCT and nasal ST, superior ST, and inferior ST (r = 0.076, 0.126, 0.183; P = 0.335, 0.238, 0.150) (Table 4).

**Table 4:** Correlation analysis between SFCT and ST in BRVO eyes.

SFCT	Temporal ST	Nasal ST	Superior ST	Inferior ST
r	0.288	0.076	0.126	0.183
*P*	0.049	0.335	0.238	0.150

*BRVO* branch retina vein occlusion, *SFCT* subfoveal choroid thickness, *ST* scleral thickness.

P < 0.05 was statistically significant.

Pearson correlation analysis revealed that in BRVO patients, there was a significant positive correlation between CRT and temporal ST (r = 0.355, P = 0.04). There was no correlation between CRT and nasal ST, superior ST, and inferior ST (r = − 0.081, − 0.102, 0.038; P = 0.650, 0.567, 0.832) (Table 5).

**Table 5:** Correlation analysis between CRT and ST in BRVO eyes.

CRT	Temporal ST	Nasal ST	Superior ST	Inferior ST
r	0.355	− 0.081	− 0.102	0.038
P	0.04	0.650	0.567	0.832

*BRVO* branch retina vein occlusion, *CRT* central retinal thickness, *ST* scleral thickness.

P < 0.05 was statistically significant.

## Discussion

Retinal vein occlusion (RVO) is the second most common retinal vascular disease in the world, following diabetic retinopathy^16^. RVO results in retinal hemorrhage, exudation, and macular edema due to retinal ischemia and hypoxia, leading to vision loss and increased societal burden. Risk factors for RVO include atherosclerosis, advanced age, hypertension, smoking, diabetes, retinal vein blood stasis, and changes in blood flow velocity^2^. However, some RVO patients do not exhibit the previously reported risk factors, indicating the involvement of other factors in RVO development.

Our study showed that the SFCT of eyes in the BRVO group was significantly increased compared to contralateral eyes and normal control group patients, with statistical significance. This finding is consistent with previous studies indicating increased SFCT in BRVO patients^17,18^. Research suggests that the pathogenic mechanisms of RVO and thick choroid diseases are similar, in RVO patients, central serous chorioretinopathy, idiopathic polypoidal choroidal vasculopathy, and other thick choroid diseases are more common, suggesting that eyes with choroidal thickening characteristics may have slowed blood flow, making them more susceptible to RVO^10^. However, it seems unlikely that the retrograde choroidal blood flow associated with localized BRVO could affect the sclera to the extent of causing overall thickening. If choroidal thickening were a cause of RVO, anti-VEGF treatment would not be expected to reduce the choroid thickness. Interestingly, studies have shown that the SFCT of eyes with primary RVO-ME is thickened. Anti-VEGF therapy can effectively reduce SFCT, improve ME, and improve BCVA^19^, suggesting that its thickening in BRVO is a consequence of increased intraocular VEGF and nitric oxide levels. Even so, the exact mechanism for choroidal thickening in BRVO are still unclear.

The choroid accounts for approximately 85% of ocular blood volume, providing oxygen, nutrients, and regulating temperature primarily to the outer retina^20^. The choroid is the most vascularized and metabolically active region in the eye, making it susceptible to influences from retinal vascular occlusions. Adequate intraocular blood circulation necessitates vessel penetration into the eye wall. Recent insights into choroidal thickness and its spectrum of diseases highlight the interplay and dependency between the three layers of the eye wall and intraocular vessels. Research has suggested that diseases involving choroidal thickness are primarily caused by choroidal vein stasis and vortex vein remodeling leading to retinal pathology, with the venous reflux within the eye likely involving the sclera^21^. Our study also demonstrates a statistically significant increase in temporal, nasal, superior, and inferior ST in BRVO-affected eyes compared to contralateral eyes and normal control group patients. Moreover, our study showed that the thickness in SFCT and temporal ST in the more pronounced venous congestion group (major BRVO) is higher than the less congestion group. This may be attributed to choroidal blood flow reflux through vortex veins, which enter the superior and inferior ophthalmic veins through the sclera. The increased thickness or hardness of the sclera may compress the vortex veins, leading to impaired choroidal blood reflux, venous stasis, ischemia, hypoxia, increased vascular endothelial growth factor, changes in vascular permeability, interstitial edema, and increased SFCT. Our speculation is consistent with previous research findings. Taiji et al. studied the SFCT in patients with RVO before and after intravitreal injection of aflibercept, showing a decrease in SFCT after the treatment^22^. Kohji et al. followed RVO patients for 12 months after intravitreal injection of anti-vascular endothelial growth factor therapy and found no statistically significant changes in retinal superficial and deep vascular densities or in the area of the avascular zone in the fovea, but observed a decrease in choroidal thickness^8^. Chung et al. demonstrated a significant increase in choroidal volume in eyes affected by BRVO compared to contralateral eyes, which decreased after intravitreal injection of anti-vascular endothelial growth factor therapy. They also suggested that the increased vascular permeability in BRVO patients is the reason for the thickening of SFCT, which decreases after treatment, leading to a reduction in vascular permeability^23^. These studies indicate that an increase in SFCT in BRVO patients is not related to changes in vascular density but may be associated with alterations in vascular permeability. Our correlation analysis in this study also revealed a positive correlation between SFCT/CRT and temporal ST in BRVO patients (r = 0.288, 0.355, P = 0.049, 0.04). There was no correlation between SFCT and nasal, superior, and inferior ST (P > 0.05). This may be attributed to the majority of cases in our study being patients with temporal branch vein occlusion, indicating a correlation between the increase in SFCT/CRT and the location of vascular obstruction in BRVO. Additionally, our study found that the presence of macular edema (ME) in BRVO did not have a significant impact on SFCT, as well as temporal, nasal, superior, and inferior ST. What’s more, our study also showed a general increase in scleral thickness and an increase in SFCT in the contralateral eyes of BRVO patients, which further confirmed the presence of scleral and choroidal thickening features in BRVO patients.

To the best of our knowledge, this is the first study investigating changes in ST in BRVO. Our study indicates an increase in SFCT, temporal ST, nasal ST, superior ST, and inferior ST in eyes affected by BRVO. However, our study also has some limitations: 1. It is a cross-sectional study, lacking longitudinal comparisons before and after intravitreal anti-vascular endothelial growth factor therapy and laser treatment in BRVO patients; 2. We did not utilize optical coherence tomography angiography to quantitatively analyze choroidal vasculature to further validate our speculations.3. The sample size was small, and due to the different pathogenic mechanisms of BRVO and CRVO^24^, our study only included BRVO patients, lacking research on CRVO patients.4. This study lacks research on the optic disc morphology and blood flow in patients with BRVO. It is currently unclear whether small optic discs are also associated with thick sclera and play a significant role in the occurrence and development of BRVO. In CRVO, a small optic disc is a risk factor, but Yang Rundong et al. measured optic disc morphological parameters, including cup-to-disc ratio, optic disc area, and rim area, in patients with unilateral RVO, contralateral eyes, and healthy control groups, and found no significant differences^25^. Zhu Shaojin et al. found a decrease in blood flow density in the optic disc region of the affected eye in patients with unilateral RVO^26^; 5. All measurements were done manually, and measurement errors were unavoidable. Taking the average value of ST at different distances from the corneal edge may reduce some measurement errors. The next step in our exploration will involve a large-sample prospective comparative study using optical coherence tomography angiography to investigate the effects of intravitreal anti-vascular endothelial growth factor therapy in patients with RVO.

## Conclusion

In patients with BRVO, both SFCT and ST are increased, and there is a significant correlation between SFCT and the location of vascular obstruction in ST.

## Data Availability

The datasets generated and/or analysed during the current study are not publicly available due but are available from the corresponding author on reasonable request.
